# Insights into the Mechanisms Involved in Strong Hemorrhage and Dermonecrosis Induced by Atroxlysin-Ia, a PI-Class Snake Venom Metalloproteinase

**DOI:** 10.3390/toxins9080239

**Published:** 2017-08-02

**Authors:** Luciana Aparecida Freitas-de-Sousa, Mônica Colombini, Mônica Lopes-Ferreira, Solange M. T. Serrano, Ana Maria Moura-da-Silva

**Affiliations:** 1 Programa de Pós-Graduação em Ciências-Toxinologia, Laboratório de Imunopatologia, Instituto Butantan, São Paulo 05503-900, Brazil; luciana.sousa@butantan.gov.br; 2 Laboratório de Imunopatologia, Instituto Butantan, São Paulo 05503-900, Brazil; monica.colombini@butantan.gov.br; 3 Laboratório Especial de Toxinologia Aplicada, Center of Toxins, Immune-Response and Cell Signalig, CeTICS, Instituto Butantan, São Paulo 05503-900, Brazil; monica.lopesferreira@butantan.gov.br (M.L.-F.); solange.serrano@butantan.gov.br (S.M.T.S.)

**Keywords:** snake, venom, metalloproteinase, dermonecrosis, hemorrhage, *Bothrops atrox*, extracellular matrix

## Abstract

Hemorrhage is the most prominent effect of snake venom metalloproteinases (SVMPs) in human envenomation. The capillary injury is a multifactorial effect caused by hydrolysis of the components of the basement membrane (BM). The PI and PIII classes of SVMPs are abundant in viperid venoms and hydrolyze BM components. However, hemorrhage is associated mostly with PIII-class SVMPs that contain non-catalytic domains responsible for the binding of SVMPs to BM proteins, facilitating enzyme accumulation in the tissue and enhancing its catalytic efficiency. Here we report on Atroxlysin-Ia, a PI-class SVMP that induces hemorrhagic lesions in levels comparable to those induced by Batroxrhagin (PIII-class), and a unique SVMP effect characterized by the rapid onset of dermonecrotic lesions. Atroxlysin-Ia was purified from *B. atrox* venom, and sequence analyses indicated that it is devoid of non-catalytic domains and unable to bind to BM proteins as collagen IV and laminin in vitro or in vivo. The presence of Atroxlysin-Ia was diffuse in mice skin, and localized mainly in the epidermis with no co-localization with BM components. Nevertheless, the skin lesions induced by Atroxlysin-Ia were comparable to those induced by Batroxrhagin, with induction of leukocyte infiltrates and hemorrhagic areas soon after toxin injection. Detachment of the epidermis was more intense in skin injected with Atroxlysin-Ia. Comparing the catalytic activity of both toxins, Batroxrhagin was more active in the hydrolysis of a peptide substrate while Atroxlysin-Ia hydrolyzed more efficiently fibrin, laminin, collagen IV and nidogen. Thus, the results suggest that Atroxlysin-Ia bypasses the binding step to BM proteins, essential for hemorrhagic lesions induced by PII- and P-III class SVMPs, causing a significantly fast onset of hemorrhage and dermonecrosis, due to its higher proteolytic capacity on BM components.

## 1. Introduction

Many pathological effects caused by snakes of the genus *Bothrops* in human envenomings are related to the direct or indirect activity of snake venom metalloproteinases (SVMPs), such as hemorrhage [1], skeletal muscle necrosis [2], skin damage [3,4], inhibition of platelet aggregation and coagulopathies [5], edema and inflammatory reactions [6,7,8]. SVMPs are zinc-dependent multi-domain proteins, which have been classified in three classes according to their domain composition in mature form. The PI-class SVMPs have only a catalytic domain (MP), whereas those of the PII-class contain a MP domain and a disintegrin domain, which is generally processed to generate free disintegrins. PIII-class SVMPs have, in addition to the MP domain, disintegrin-like and cysteine-rich domains (DC) [9]. Hemorrhage is a very important effect induced by SVMPs and is directly correlated to the hydrolysis of the basement membrane (BM) proteins from micro vessels [10,11,12]. Three factors are important for this effect: the binding of the enzymes to the BM components of the capillaries [11]; the localization at BM enhancing the hydrolysis of BM and cell proteins involved in capillary stability and cell anchorage [13]; and the hemodynamic forces within the microcirculation contribute to the distension and rupture of the capillary wall, with consequent hemorrhage [10].

SVMPs are generally capable of degrading BM components in vitro, however, only particular types of SVMPs are able to induce hemorrhage in vivo [12,14]. Baldo et al. [11] and Herrera et al. [15] demonstrated that PII-class and PIII-class SVMPs accumulated in the BM due to specific binding of their DC domains to collagen IV, thus facilitating the hydrolysis and consequent hemorrhage. Conversely, the PI-class SVMPs would not be able to bind to BM molecules and as a consequence would spread through the tissue explaining their reduced ability to generate hemorrhage. 

Furthermore, there are marked differences between enzymes of the PI-class SVMPs isolated from *Bothrops* venoms in their ability to cause hemorrhage. For example, Neuwiedase from *B. neuwiedi* [16], BjussuMP-II from *B. jararacussu* [17], BmooMP-α1 from *B. moojeni* [18] and Leucurolysin-A from *B. leucurus* [19] do not induce any hemorrhage. On the other hand, BpirMP from *B. pirajai* [20], BmHF-1 from *B. marajoensis* [21], BaP1 from *B. asper* [22], and Batroxase from *B. atrox* [23] are able to induce certain levels of hemorrhage exhibiting minimal hemorrhagic dose (MHD) of 10–50 µg/mice, which is very high compared to the MHD of PII-class and PIII-class SVMPs that is frequently below 1 µg/mice. However, many issues about the mechanism of hemorrhage induction by PI-class SVMPs still need to be resolved.

In a recent study, we isolated and characterized the major PIII-class SVMP (Batroxrhagin) from *B. atrox* venom [24]. *B. atrox* is considered to be the major cause of snakebite in the Amazon region [25] and the symptoms of human envenoming by *B. atrox* are strongly correlated with the high abundance of PIII-class SVMPs in this venom [26,27]. Following the protocol for Batroxrhagin purification, we detected a highly hemorrhagic fraction that also caused necrosis in the mouse skin, and, interestingly, contained proteins with the molecular masses of PI-class SVMPs. In this study, we isolated and characterized a highly hemorrhagic P-I SVMP, named Atroxlysin-Ia, and describe its hemorrhagic and dermonecrotic activities in comparison to Batroxrhagin, a typical hemorrhagic PIII-class SVMP from the same venom. Our data indicate that a high hydrolytic activity of Atroxlysin-Ia towards extracellular matrix (ECM) proteins, including laminin, might explain its ability to cause a fast disruption of capillary vessels and detachment of epidermis layers, allowing the induction of hemorrhage and dermonecrosis, despite the absence of non-catalytic domains in the enzyme.

## 2. Results

### 2.1. Purification and Identification of the Major PI-Class SVMP of B. atrox Venom

**Figure 1:**
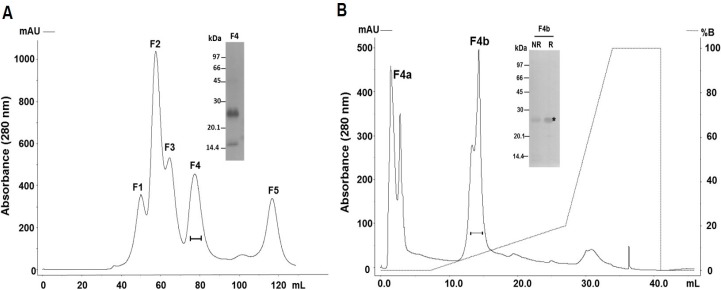
Purification of the PI-class SVMP from *B. atrox* venom. Venom samples (50 mg) were applied to HiPrep 16/60 Sephacryl S-200 HR column and eluted at flow rate of 0.5 mL/min in 20 mM Tris/HCl buffer, pH 7.8, containing 150 mM of NaCl (**A**). Fractions containing ~25 kDa proteins presenting hemorrhage and dermonecrosis activities (continuous line under the peaks) were pooled, applied into a Mono Q 5/50 GL anion exchange column and eluted at flow rate of 1 mL/min with a gradient from 0 to 1 M of NaCl (**B**). Pooled fractions (10 µg) indicated in the graphs by continuous line under the peaks were subjected to SDS-PAGE under reducing (R) or non-reducing conditions (NR) and silver-stained. The band indicated by * was excised and submitted to MS/MS.

**Figure 2:**
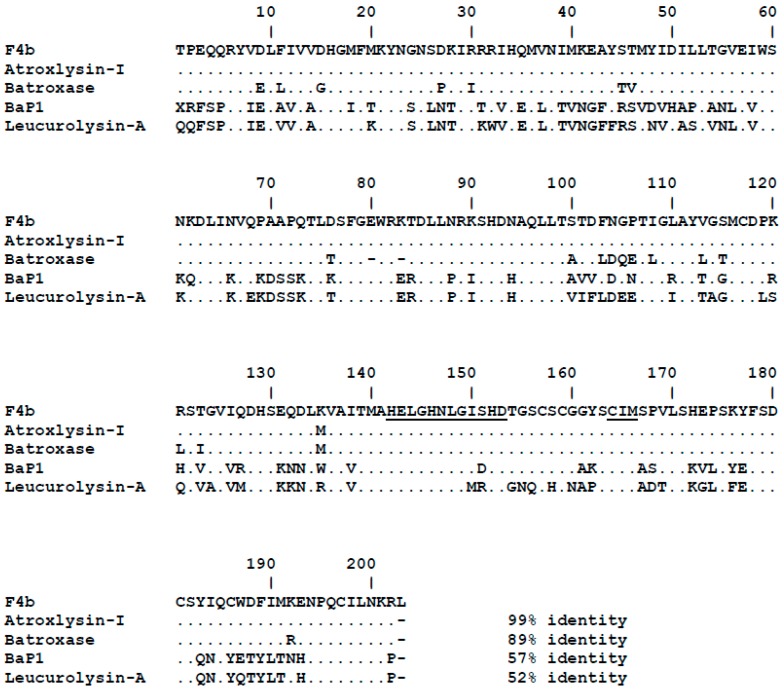
Sequence alignment of the isolated toxin with snake venom metalloproteinases (SVMPs) from different species of *Bothrops*. Amino acid sequence of the protein isolated in the fraction F4b was aligned with PI-class SVMPs: Atroxlysin-I (P85420) and Batroxase [23] from *B. atrox* venom, BaP1 (P83512) from *B. asper* venom and Leucurolysin-A (P84907) from *B. leucurus* using ClustalW [30]. Dots indicate identical residues to the first sequence. Functional motifs corresponding to the zinc-binding and Met-Turn are underlined.

The purification of a PI-class SVMP from *B. atrox* venom was carried out by size-exclusion and anion-exchange chromatographies (Figure 1). In size exclusion chromatographies, venom samples (50 mg) were fractionated in five major peaks (Figure 1A) and fractions concentrating 25 kDa proteins and showing hemorrhagic and dermonecrotic activities (continuous line under the peak F4) were pooled, applied into an anion-exchange column and eluted in two peaks both presenting bands of approximately 25 kDa (Figure 1B). However, only fraction F4b showed preserved dermonecrotic activity and was recovered as our target protein with a purification yield of approximately 4%. The fraction F4b was resolved in SDS-PAGE as a band of the 25 kDa under non-reducing or reducing conditions (Figure 1B), which was excised from the gel and submitted to mass spectrometric protein identification. The tryptic peptide sequences, identified using a *B. atrox* venom gland transcriptome database, are summarized in Table 1 and match the complete sequence corresponding to the mature protein of a single abundant transcript of *B. atrox* venom gland (BATXSVMPI5). Figure 2 shows the sequence alignment of the isolated protein with other PI-class SVMPs: Atroxlysin-I [28] and Batroxase [23] from *B. atrox* venom, BaP1, a hemorrhagic PI-class SVMP from *B. asper* venom [29] and Leucurolysin-A, a non-hemorrhagic PI-class SVMP from *B. leucurus* venom [19]. The isolated SVMP showed 99% identity with Atroxlysin-I, with only one substitution at residue 135 (methionine for lysine), 89% with Batroxase, 57% with BaP1 and 52% with Leucurolysin-A. Thus, based on the high identity with Atroxlysin-I, we concluded that we isolated an isoform of this toxin and in the rest of the work the isolated PI-class SVMP (F4b) will be called Atroxlysin-Ia.

### 2.2. Hemorrhagic and Dermonecrotic Activities of Atroxlysin-Ia

**Figure 3:**
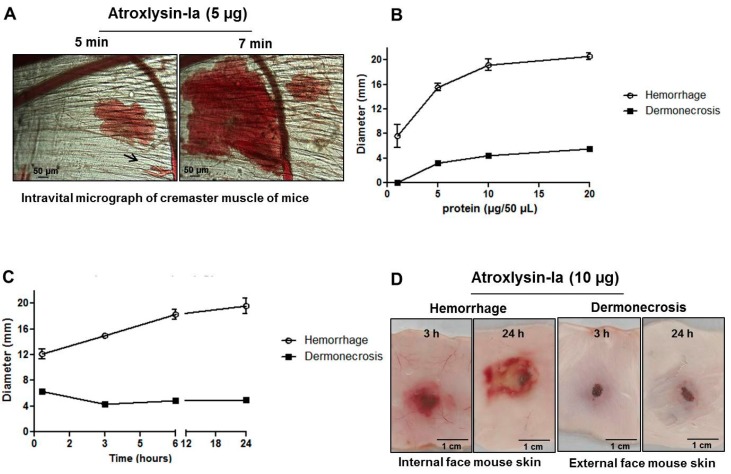
Hemorrhagic and dermonecrotic activities of Atroxlysin-Ia. (**A**) Intravital micrograph of mice cremaster muscle after local administration of Atroxlysin-Ia (5 µg) evidencing the rupture of the capillary vessels indicated by arrow. (**B**) Size of hemorrhagic and dermonecrotic lesions induced by different doses of Atroxlysin-Ia, evaluated at 24 h after the intradermal injection. (**C**) The progression of the lesions evaluated at 20 min, 3, 6 and 24 h after injection of 10 µg Atroxlysin-Ia. (**D**) Macroscopic view of hemorrhagic (inner dorsal skin) and dermonecrosis (outer dorsal skin) activities evaluated at 3 h and 24 h after the intradermal injection of 10 µg Atroxlysin-Ia. (**A**,**D**) Figures are representative of three independent mice. (**B**,**C**) Results are expressed as mean and SEM of three independent experiments.

**Figure 4:**
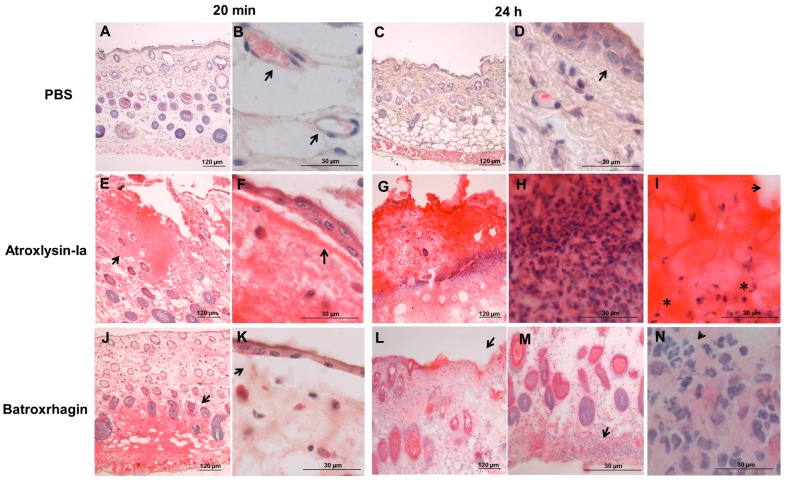
Action of Atroxlysin-Ia and Batroxrhagin in the skin of mice. Histological analysis (hematoxylin and eosin staining of paraffin embedded sections) of the site of injection of 10 μg of toxins. The group injected with phosphate-buffered saline (PBS) showed the normal histological pattern of skin (**A**,**C**) with preserved capillaries (arrow in **B**) and the undamaged epidermis (arrow in **D**). Tissues injected with Atroxlysin-Ia showed hemorrhage especially in the epidermis region (arrow in **E**) and Batroxrhagin induced hemorrhage predominantly in the hypodermis region (arrow in **J**). Both toxins induced epidermis detachment (arrows in **F**,**K**). Atroxlysin-Ia induced a large lesion in the epidermis and dermis (**G**), which evolved to the necrosis of the tissue characterized by exacerbated inflammatory infiltrate (**H**), deposit of fibrin clot (arrow in **I**) and pycnotic nuclei of the cells (* in **I**). Batroxrhagin induced a loss of epidermis (arrows in **L**) and inflammatory infiltrate especially in the hypodermis region (arrows in **M**,**N**).

Figure 3 and Figure 4 show the characterization of the hemorrhagic and dermonecrotic activities of Atroxlysin-Ia. Figure 3A shows the establishment of the hemorrhagic process, in particular the rupture of the capillary wall (arrow) detected by intravital microscopy of mice cremaster muscle at 5 min after the topical application of 5 µg of Atroxlysin-Ia. Hemorrhage and dermonecrosis induced by Atroxlysin-Ia presented a dose-dependent pattern (Figure 3B), while only hemorrhage shows a pattern of time response, whereas dermonecrosis appears to be a rapidly triggered process and does not evolve to a bigger area after 20 min (Figure 3C). The macroscopic view of the internal and external face of the mice skin is shown in Figure 3D evidencing the hemorrhage and dermonecrosis at 3 and 24 h after injection of 10 µg Atroxlysin-Ia. In addition, hemorrhagic and dermonecrotic activities of Atroxlysin-Ia were evaluated in the presence of metalloproteinase and serine proteinase inhibitors. Both hemorrhagic and dermonecrotic activities were inhibited by 1,10-Phenanthroline, a Zn(2+)-chelating metalloproteinase inhibitor, but not by Phenylmethylsulfonyl fluoride, a serine proteinase inhibitor (data not show), indicating that these activities are dependent on the metalloproteinase catalytic activity of Atroxlysin-Ia.

Considering Atroxlysin-Ia high hemorrhagic activity and its ability to induce dermonecrosis, we next proceeded to the investigation of mechanisms involved in these effects by comparing the morphological alterations induced by Atroxlysin-Ia and Batroxrhagin. Because hemorrhage and skin damage induced by Atroxlysin-Ia are effects of rapid onset, we selected the time of 20 min after injection to focus on the direct action of the toxins and that of 24 h after injection to evaluate the cutaneous lesion after local reaction. Paraffin embedded sections were prepared from the sites injected with 10 μg of Atroxlysin-Ia or Batroxrhagin, and the histological analysis of these sections were carried out by hematoxylin and eosin staining. The group injected with phosphate-buffered saline (PBS) showed the normal histological pattern of skin (Figure 4A,C), and arrows indicate the preserved blood capillaries (Figure 4B) and the undamaged epidermis (Figure 4D). Tissues injected with Atroxlysin-Ia (Figure 4E,F) showed hemorrhage especially in the epidermis region (arrow in Figure 4E), and a marked epidermis detachment followed by an extensive lesion in the epidermis and dermis as soon as 20 min after injection (arrow in Figure 4F). Batroxrhagin-induced hemorrhage was concentrated in the hypodermis region (arrow in Figure 4J) and characterized by reduced levels of epidermis detachment (arrow in Figure 4K). After 24 h the group injected with Atroxlysin-Ia presented a large area of necrosis in epidermis/dermis region (Figure 4G), exacerbated inflammatory infiltrate around the lesion (Figure 4H), evidences of fibrin clots (arrow), and (*) cells with pycnotic nuclei (Figure 4I). Batroxrhagin did not show such signs of tissue necrosis at the injected region, presenting only a few regions of epidermis loss (arrows in Figure 4L) and inflammatory infiltrate (arrows in Figure 4M,N) concentrated in the hypodermis. 

Despite the strong hemorrhagic activity of Atroxlysin-Ia, it did not induce relevant myotoxic activity as the serum levels of creatine-kinase activity were not increased in mice at 3 h after intramuscular injection of the toxin (data not show).

### 2.3. Binding of SVMPs to Plasma and Extracellular Matrix Proteins In Vitro

**Figure 5:**
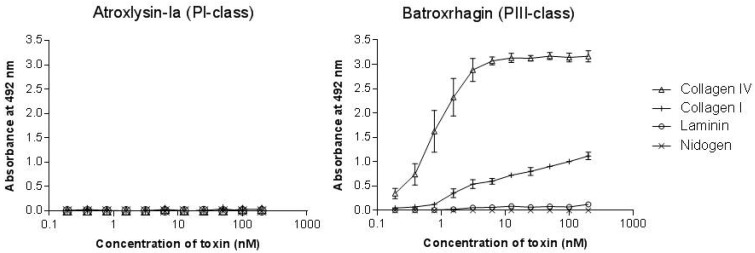
Binding of Atroxlysin-Ia and Batroxrhagin to extracellular matrix components. Microtiter plates were coated with type I and IV collagens, laminin and nidogen for 18 h at 4 °C. Different dilutions of toxins were then added to the plates and the binding was detected at 492 nm after incubation with anti-Batroxrhagin serum produced in mice, followed by anti-mouse IgG peroxidase conjugates and the enzyme substrate. The results correspond to the mean and SEM of three independent experiments.

The binding to the components of the extracellular matrix and basement membrane of the capillaries through adhesive domains is considered as the first and fundamental step in the onset the hemorrhage induced by P-II and P-III classes SVMPs. Thus, to explain the strong hemorrhage generated by Atroxlysin-Ia, we hypothesized that it would bind to BM proteins by a distinct motif present in its catalytic domain. To test this hypothesis, we first evaluated the binding to fibrinogen, fibronectin, collagens I and IV, laminin and nidogen in solid-phase binding assays. Atroxlysin-Ia was not able to bind to any ECM or plasma protein tested in contrast to the high affinity binding of Batroxrhagin to collagen I and collagen IV (Figure 5), once more suggesting that this activity is limited to motifs present in non-catalytic DC domains.

**Figure 6:**
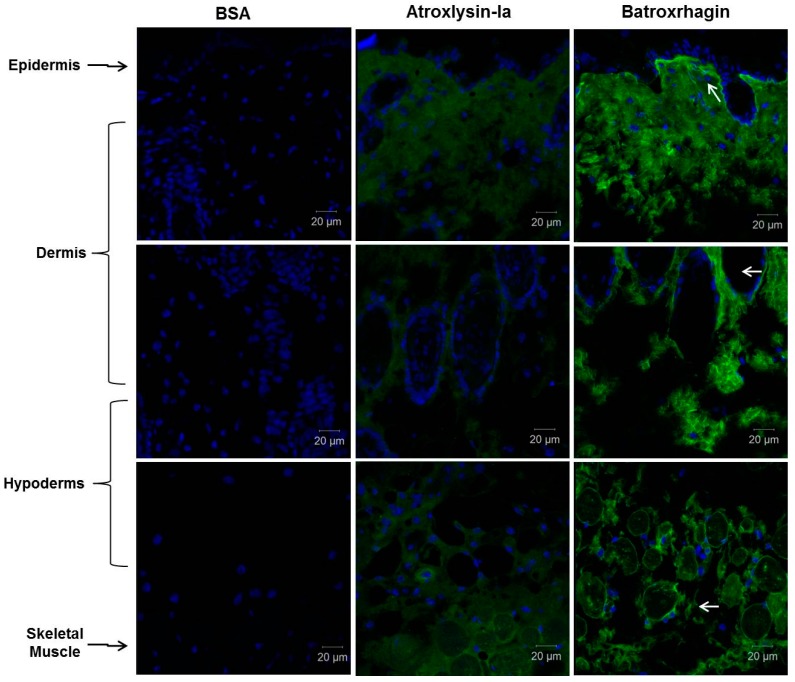
Distribution of Atroxlysin-Ia and Batroxrhagin in the skin. Cryosections were obtained at 20 min after injection of 10 µg in mice skin. Toxins and BSA (control) were labeled with Alexa488 emitting green fluorescence. Batroxrhagin accumulated in the basement membrane of epidermis, skin structures and skeletal muscle cells (arrows). Atroxlysin-Ia shows a weaker diffuse fluorescence and BSA did not show any fluorescence. The nuclei of the cells were stained in blue (4′,6-diamidino-2-phenylindole) and sections were examined with a confocal microscope.

**Figure 7:**
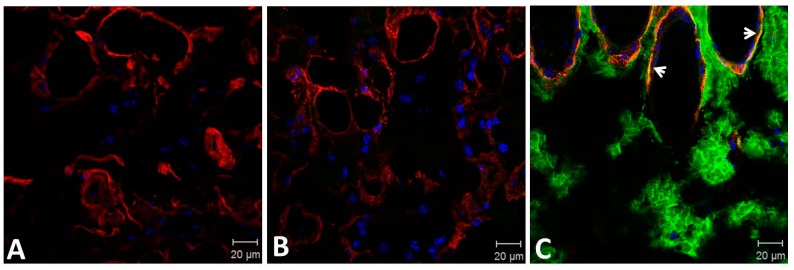
Co-localization of Atroxlysin-Ia and Batroxrhagin with basement membrane proteins in the skin. Cryosections were obtained at 20 min after intradermal injection of 10 μg in mice skin. BSA (**A**), Atroxlysin-Ia (**B**) and Batroxrhagin (**C**) were labeled with Alexa488 emitting green fluorescence. The laminin was stained with rabbit polyclonal anti-laminin labelled with Tetramethyl Rhodamine Isothiocyanate (red). Batroxrhagin co-staining with laminin is represented by yellow dots (arrows in **C**). The nuclei of the cells were stained blue (4′,6-diamidino-2-phenylindole) and sections were examined with a confocal microscope.

Tissue-binding of Atroxlysin-Ia was also evaluated in vivo in comparison to Batroxrhagin. Initially, distribution of toxins labeled with Alexa488 in the skin extension of the mice was evaluated by confocal microscopy at 20 min after injection (Figure 6). Atroxlysin-Ia showed a weak and diffuse fluorescence that accumulated more in the epidermis/dermis region. By contrast, Batroxrhagin was distributed throughout the skin’s extension and concentrated near the structures of the appendages of the skin including the skeletal muscle adjacent to the hypodermis in which Batroxrhagin appeared with a higher fluorescence mainly around the bundles of muscle fibers. The mice which received bovine serum albumin instead of toxins (BSA) did not show any fluorescence in the analyzed regions. Batroxrhagin but not Atroxlysin-Ia was co-localized with BM proteins as observed by co-staining with laminin represented by yellow dots in Figure 7C. Taken together, in vitro and in vivo experiments demonstrated that the strong hemorrhage induced by Atroxlysin-Ia is independent of binding to ECM proteins and therefore, follows a mechanism that does not involve the steps which are fundamental for PIII-class SVMP-induced hemorrhage.

### 2.4. Hydrolytic Activity of Hemorrhagic SVMPs In Vitro

**Figure 8:**
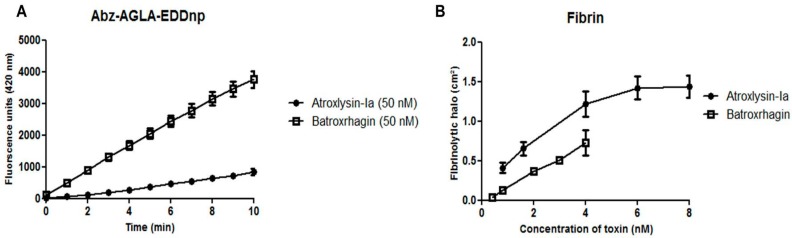
Hydrolytic activity of the Atroxlysin-Ia and Batroxrhagin in vitro. (**A**) Hydrolysis of synthetic peptide Abz-AGLA-EDDnp was assayed by fluorimetric assays using 200 nM of the Abz-AGLA-EDDnp peptide and 50 nM of toxins at 37 °C monitored by fluorescence at λEM 420 nm and λEX 320 nm using a spectrofluorometer. (**B**) The fibrinolytic activity was carried out in solidified fibrin-agarose gels. Different concentrations of the toxins were applied in the gel and the hydrolysis area was measured after incubation for 18 h at 37 °C. Results are expressed as the area of lysis (cm^2^). The results are the mean and standard error (SEM) from three independent experiments.

Since Atroxlysin-Ia showed no binding to BM components, we decided to evaluate if differences in catalytic activity could explain the high levels of hemorrhage observed after injection of the toxin. For this purpose, we compared Atroxlysin-Ia and Batroxrhagin catalytic activities towards distinct substrates. Batroxrhagin hydrolyzed more efficiently than Atroxlysin-Ia a peptidic synthetic substrate (Abz-AGLA-EDDnp), while Atroxlysin-Ia hydrolyzed fibrin more efficiently (Figure 8A,B, respectively). Furthermore, due to the importance of degradation of basement membrane components in the hemorrhage induction process, we compared the hydrolysis of these components in vivo and in vitro.

**Figure 9:**
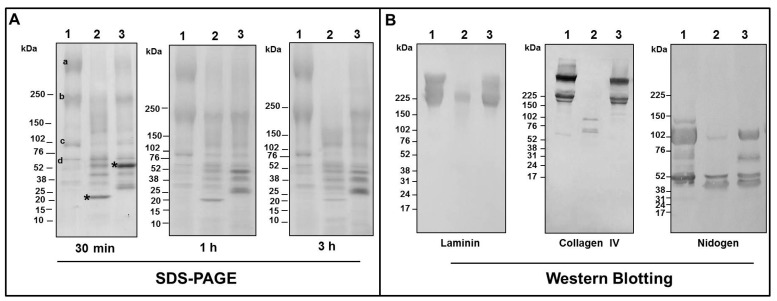
Hydrolysis of the basement membrane components by Atroxlysin-Ia and Batroxrhagin in vitro. (**A**) Matrigel (50 µg) was incubated with 8 µM of each toxin at 37 °C and the products of hydrolysis were evaluated by SDS-PAGE under reducing conditions. (**B**) Matrigel (50 µg) was incubated with 10 µg of each toxin (Atroxlysin-Ia 16 µM and of Batroxrhagin 8 µM) and the products of hydrolysis of the laminin, collagen IV and nidogen were evaluated by Western Blotting using specific antibodies to each basement membrane (BM) protein. Molecular mass markers are indicated on the left. Experimental groups are: 1—Matrigel (control); 2—Atroxlysin-Ia + Matrigel; 3—Batroxrhagin + Matrigel. The most abundant components in the control correspond to laminin α1 chain and one chain of collagen IV (a), laminin β1 and γ1 chains and the second chain of collagen IV (b) and nidogen chains (c and d) and toxin bands are marked with *.

In the in vitro assays, the hydrolysis of the BM components was executed by incubation of Atroxlysin-Ia and Batroxrhagin with Matrigel, a reconstituted basement membrane preparation, which contains laminin as a major component, collagen IV, heparan sulfate proteoglycan, nidogen and other minor components. Figure 9A shows the kinetics of Matrigel degradation using 8 μM of each enzyme evaluated by SDS-PAGE. The control lane shows the non-hydrolysed Matrigel with prominent bands of laminin (~400 and 225 kDa, a and b, respectively), collagen IV (~400 and ~225 kDa, a and b, respectively) and nidogen (~102 and 52 kDa, c and d, respectively). The band of ~400 kDa was hydrolyzed by both SVMPs, however hydrolysis by Atroxlysin-Ia was faster and degradation was complete in 3 h of incubation. Hydrolysis of 225 kDa band by Atroxlysin-Ia was complete within 3 h while hydrolysis by Batroxrhagin was not complete to the end of the experiment. The band of ~102 kDa was not fully depleted within 3 h incubation with both metalloproteinases. Next, we focused on the specific hydrolysis of laminin, collagen IV and nidogen, by Western blotting of products generated during a 30 min incubation of matrigel and the SVMPs. Figure 9B, control lane, shows the intact laminin chains as two major bands, the first corresponding to the α1 chain (~400 kDa) and the second composed by the β1 and γ1 chains (~225 kDa). After incubation with Atroxlysin-Ia (lane 2), the α-chain was completely degraded and substantial hydrolysis of the other two chains was also observed, whereas Batroxrhagin partially hydrolyzed all the chains but not to the same extent. Both collagen IV chains (~225 and ~400 kDa) were completely hydrolyzed by Atroxlysin-Ia, resulting in two visible fragments of 52 kDa and 102 kDa, while incubation with Batroxrhagin resulted again in a weaker degradation. Regarding nidogen, the bigger chains were hydrolyzed by both enzymes, generating a fragment ~40 kDa, however, the efficiency of Atroxlysin-Ia hydrolysis was higher and differed from that of Batroxrhagin, which generated a fragment ~60 kDa.

**Figure 10:**
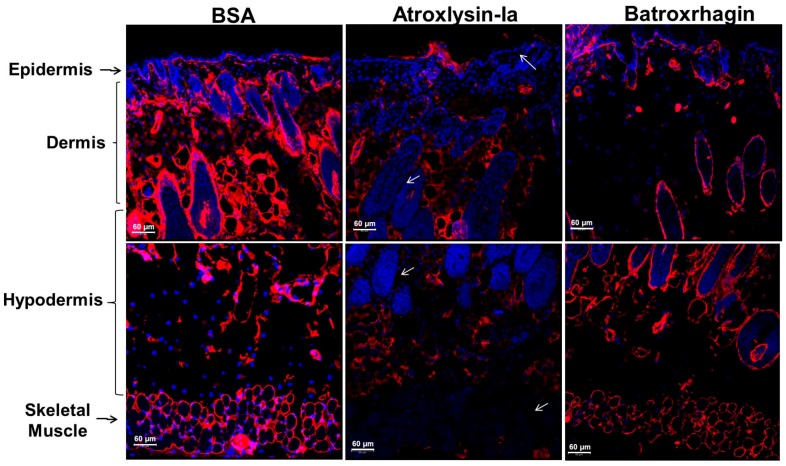
Hydrolysis of the laminin by Atroxlysin-Ia and Batroxrhagin in vivo. Cryosections were obtained at 20 min after injection of 10 µg of toxins in mice skin. Laminin is stained in red by rabbit polyclonal anti-laminin labelled with Tetramethyl Rhodamine Isothiocyanate and the nuclei of the cells was stained blue (4′,6-diamidino-2-phenylindole). The control group (BSA) show the normal distribution of laminin in the BM of the epidermis/dermis interface, around the appendages of the skin and skeletal muscle. Atroxlysin-Ia hydrolyzed more laminin in BM (arrows) than Batroxrhagin. Sections were examined with a Confocal Microscope.

**Figure 11:**
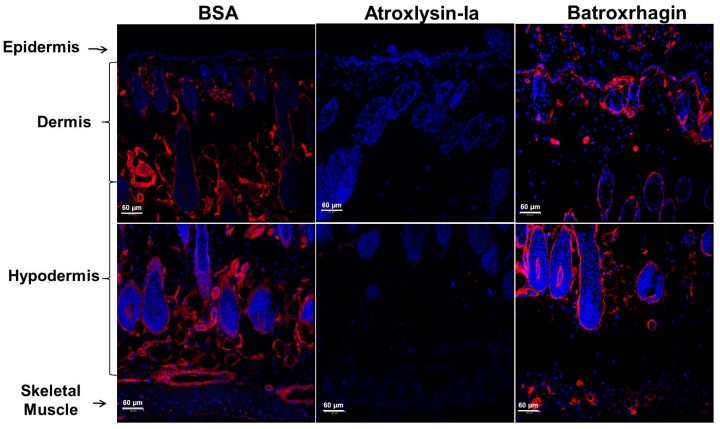
Hydrolysis of the collagen IV in the skin of mice by Atroxlysin-Ia and Batroxrhagin in vivo. The cryosections were obtained at 20 min after injection of 10 µg of toxins in mice skin. Collagen is stained in red with rabbit polyclonal anti-collagen IV labelled with Tetramethyl Rhodamine Isothiocyanate and the nuclei of the cells was stained blue (4′,6-diamidino-2-phenylindole). The control group (BSA) show the normal distribution of collagen IV in the BM of the epidermis/dermis interface, around the appendages of the skin and skeletal muscle and Atroxlysin-Ia hydrolyzed more collagen IV in BM than Batroxrhagin. Sections were examined with a Confocal Microscope.

The differences between Atroxlysin-Ia and Batroxrhagin in their proteolytic potency in vitro were significant, so we decided to evaluate the process in vivo. The hydrolysis of the BM components was analyzed by immunodetection of laminin and collagen IV in the skin of mice after injection of the toxins. In Figure 10 and Figure 11, the normal distribution of collagen and laminin in the BM of the epidermis/dermis interface, around the appendages of the skin and skeletal muscle can be observed in the control group (BSA). After injection of the toxins, discrete hydrolysis of collagen and laminin was observed in tissues treated with Batroxrhagin. In contrast, Atroxlysin-Ia hydrolyzed laminin and collagen IV immunolocalized in BM of the epidermis/dermis interface, around the appendages of the skin and skeletal muscle (arrows in Figure 10 and Figure 11). In summary, in vitro Atroxlysin-Ia degraded laminin and collagen IV faster and more efficiently than Batroxrhagin. In in vivo experiments, collagen IV and laminin were depleted in the group injected with Atroxlysin-Ia. These results suggest that the higher catalytic activity of Atroxlysin-Ia towards ECM proteins bypasses the binding to ECM, a crucial step for hemorrhage induced by PIII SVMPs.

## 3. Discussion

In this paper we describe unique activities of a PI-class SVMP isolated from *Bothrops atrox* venom, Atroxlysin-Ia, that induces strong hemorrhage and dermonecrosis in the skin of mice. Other PI-class SVMPs have already been isolated from *B. atrox* venom as HI-5 [31], Batx-I [32] and Batroxase [23,33]. Batroxase showed 89% identity with Atroxlysin-Ia, while HI-5 and Batx-I have not a complete amino acid sequence and thus alignment was not possible. Alignment of the sequence obtained with other PI-class SVMPs, purified from the *B. atrox* venom, revealed that the toxin isolated in this work is an isoform of Atroxlysin-I already isolated from *B. atrox* venom originating from snakes from Peru [28]. Interestingly, all these PI-class SVMPs were considered weakly hemorrhagic, and no induction of dermonecrosis was described in the former studies. Although the difference observed between Atroxlysin-Ia and the previously isolated Atroxlysin-I is only one amino acid, in the previous study [28] the ability of Atroxlysin-I to induce dermonecrosis have not been reported and the MHD was much higher than the MHD reported here for Atroxlysin-Ia. Some hypotheses may be upraised to explain this difference in hemorrhagic activity. The presence of post-translational modifications such as the glycosylation pattern may contribute to the complexity of the venom [34]. In this sense, it has already been demonstrated that the N-glycosylation is very important for the hemorrhagic activity of SVMPs from *B. jararaca* venom [35]. However, we searched for putative *N*-glycosylation sites in the Atroxlysin-Ia sequence and no evidence to support this hypothesis was found. Perhaps, despite the similarity in the purification protocols, some steps may have led to the decrease/loss of Atroxlysin-I activity during the first purification process [28]. Moreover, differences in animal strains or experimental procedures may have masked the peculiar hemorrhagic and dermonecrotic activities of Atroxlysin-I when it was first isolated. As described here, hemorrhage caused by Atroxlysin-Ia was comparable to hemorrhage induced by PIII-class SVMPs. Atroxlysin-Ia MHD was 2.2 μg, which is very similar to the values detected for the PIII-class SVMPs Batroxrhagin (1.4 µg/mice) [24] and Jararhagin (1.5 µg/mice) [36] and much higher than the MHD observed for most of the other PI-class SVMP described in the literature ranging from 10 to 50 µg/mice [20,21,22,23], besides many others that do not cause hemorrhage at all [17,18,19]. 

The hemorrhagic process and skin damage are associated with the hydrolysis of the components of the basement membrane [4,10,11]. The mechanisms of hemorrhage induced by SVMPs currently accepted in the literature are dependent on non-catalytic domains, which would facilitate hydrolysis of PII and PIII-class by binding with high affinity to these components. In several activities of SVMPs, such as inhibition of platelet aggregation, the non-catalytic domains play an important role in the recognition and selectivity of substrates [37]. However, in this work we describe that Atroxlysin-Ia, devoid of such domains, shows hemorrhagic activity similar to a PIII-class SVMP. This observation indicates that a different mechanism modulates Atroxlysin-Ia-induced hemorrhage. Thus, we initially hypothesized that other binding sites present in the metalloproteinase domain could be responsible for adhesion with targets in the basement membrane. To test this hypothesis, we carried out in vitro and in vivo binding assays using the different components of extra cellular matrix (ECM). In our experiments, P-III class SVMP Batroxrhagin was able to bind to collagen IV, in vitro and in vivo, and also co-localized with laminin in vivo in a similar fashion as previously described for Jararhagin, a PIII-class SVM with high level of identity with Batroxrhagin, which is able to bind to collagen IV [11,38,39]. In contrast, Atroxlysin-Ia did not bind to any tested ECM components and presented only diffuse tissue-distribution after injection in mice skin. These results reinforce that the binding to BM components is an important part of induction of hemorrhage by PIII-class SVMPs, nevertheless, it is not involved in the mechanism of Atroxlysin-Ia action.

Hydrolysis of the basement membrane components has been ascribed as the key mechanism responsible for SVMPs hemorrhagic activity. Thereby, the next step was to analyze the hydrolytic activity of Atroxlysin-Ia and Batroxrhagin using different substrates. In the in vitro assays, Batroxrhagin was more active in the hydrolysis of a synthetic peptide substrate (Abz-AGLA-EDDnp) and Atroxlysin-Ia was more active in the hydrolysis of macromolecular substrates, such as fibrin, and induced almost complete hydrolysis of laminin, collagen IV and nidogen assembled in Matrigel. In the in vivo experiments, both toxins degraded laminin and collagen IV, although Atroxlysin-Ia has totally depleted collagen IV at the lesion site. In a different study, the catalytic activity of BaP1, considered as weakly hemorrhagic, and Leucurolysin-A, devoid of hemorrhagic activity was compared [12]. Both BaP1 and Leucurolysin-A showed a similar pattern of hydrolysis of laminin, nidogen and perlecan in vitro, although collagen IV was digested in vivo only by BaP1. The hydrolysis of collagen IV by SVMPs is attributed as the main event for the development of bleeding through the rupture of the capillaries [11,12,15,40]. The degradation of collagen IV in vivo by Jararhagin, a hemorrhagic PIII-class SVMP, and BnP1, a very weakly hemorrhagic PI-class SVMP, has also been compared showing marked degradation of collagen IV by Jararhagin [11]. Our results are in agreement with the role of collagen IV hydrolysis as a crucial point to generate hemorrhage, since both toxins degrade this component very well in vivo. In addition, hydrolysis of laminin may be a contributory factor to Atroxlysin-Ia-induced hemorrhage. Collagen IV and laminin individually self-assemble into superstructures and these networks are very important for BM stability [41,42]. The laminin and collagen IV networks are connected by nidogen and perlecan bridge and thus influence the structural integrity of BM [43]. Atroxlysin-Ia depleted almost entirely the laminin present in the basal lamina below the epidermis. Although Batroxrhagin was co-localized with laminin in that region, the extent of degradation was lower than in the case of Atroxlysin-Ia.

Based on these results we reinforce that the hydrolysis of BM components, especially collagen IV, acts as a key factor for the induction of hemorrhage. Although Atroxlysin-Ia does not exhibit DC domains and is not be able to co-localize with BM components in the skin, it is able to deplete collagen IV and hydrolyze laminin in vivo. Our data suggest that Atroxlysin-Ia bypasses the binding step characteristic of the PIII-class SVMPs due to its high catalytic activity towards BM proteins. In the literature, differences were described in the region close to the active site, which could explain this discrepancy in activities between SVMPs. The region which comprises residues 153–176, could influence the interaction with substrates of the ECM [44,45]. In addition, hemorrhagic PI-class SVMPs exhibit extensive acidic aminoacid spots, which would facilitate the attraction of positive charges of the substrates [46]. In a recently published article [47], the authors compare two dynamical models of Atroxlysin-I with Leucurolysin-A, considered in this study as examples of hemorrhagic and non-hemorrhagic PI-class SVMP respectively. The analysis revealed some changes in local structure, surface and flexibility that could contribute to explain the differences in activity between the two toxins. The region corresponding to residues 149–178 (long Ω-loop) is more flexible in Atroxysin-I, and, in addition, marked differences were observed in the surface electrostatic potential around the active site. Thus, probably this region of Atroxlysin-Ia around the active site, that presents more flexibility and greater electrostatic potential, is facilitating the attraction and anchoring of the substrates of the ECM. In this way, it would explain in part the high catalytic activity observed in this work, but more in-depth studies regarding the specificity to BM substrates are necessary to better understand the higher catalytic activity of Atroxlysin-Ia compared to other weakly-hemorrhagic PI-class SVMPs.

Atroxlysin-Ia also induced necrosis in mice skin in a very peculiar fashion. First, necrosis was rapidly established inducing a detachment of the epidermis layer within 20 min. Interestingly, the lesion did not increase macroscopically in a time-dependent pattern. However, drastic and more extensive alterations were microscopically observed, characterized by loss of epidermis, necrosis of the dermis, strong inflammatory infiltrate and formation of eschar after at 24 h after an injection. The minimum necrotizing dose (MND) of Atroxlysin-Ia was of 1.8 µg when analyzed at 24 h after the injection, which is a dose much smaller than those observed for any other PI- or PIII-class SVMPs. One reason why the skin lesion does not increase macroscopically could be related to the fast diffusion of the toxin, as it does not exhibit the adhesive domains present in the PIII-class SVMPs to localize in the tissue. This may be supported by results showing the diffuse distribution of Atroxlysin-Ia in the skin after injection of Alexa-488-labeled toxin, while Batroxrhagin was co-localized with the basement membrane. One hypothesis to explain the unique dermonecrotic action of Atroxlysin-Ia could be its efficacy in laminin hydrolysis. In other studies reporting epidermal damages as human junctional epidermolysis bullosa [48] it was shown that mutations in the laminin molecule result in impaired anchorage and detachment of the epidermis, similar to the lesions described here.

The identification of a potent dermonecrotic factor in *B. atrox* venom has a clinical implication explaining why some patients bitten by *B. atrox* present more severe local lesions. Moreover, dermonecrosis induced by *B. atrox* venom in mice is not neutralized by antivenom, except if the antivenom is administrated soon after venom injection [49]. Thus, our results are important since they indicate that dermonecrosis is a direct effect of toxins and Atroxlysin-Ia may contribute greatly to the patient’s local condition thus deserving special attention for its neutralization by antivenoms.

In conclusion, we propose that a different mechanism is responsible for the induction of hemorrhage by Atroxlysin-Ia. While PIII-class SVMPs cause hemorrhage primarily due to their adhesive capacity to collagen IV and laminin, Atroxlysin-Ia bypasses the binding step due to its high hydrolyzing activity on the components of the basement membrane, mainly laminin and collagen IV. Laminin degradation may be the key factor in the development of dermonecrosis observed in the skin of mice and the hydrolysis of collagen IV may be the main event causing hemorrhage. Therefore, with these data, we describe another mechanism of induction of hemorrhage based mainly on a high catalytic activity specific to ECM components.

## 4. Materials and Methods

### 4.1. Venom

*B. atrox* venom pool was obtained by Herpetology Laboratory, Instituto Butantan, São Paulo, Brazil. The venom was extracted from snakes maintained in captivity at Herpetology Laboratory, collected in distinct States of the Brazilian Amazon and maintained for more than 10 years in captivity under controlled light and temperature and fed with rodents. The IBAMA license for Management Authorization of Wildlife was 3550.6429/2012-SP. The venom was lyophilized and stored frozen until use. 

### 4.2. Animals

Swiss mice of both sexes (18–20 g) were obtained from the central animal house of Instituto Butantan. The animal protocols used in this work were evaluated and approved by the Animal Use and Ethic Committee (CEUAIB) of the Instituto Butantan (Protocol 1270/14). They are in accordance with COBEA guidelines and the National law for Laboratory Animal Experimentation (Law No. 11.794, 8 October 2008).

### 4.3. Purification of SVMPs

The PIII-class SVMP named Batroxrhagin was isolated from *B. atrox* venom was isolated as previously described [24], and used for comparison of biological activities. The PI-class SVMP was isolated by size exclusion and ion exchange chromatographies (AKTA explorer 10S, GE Healthcare, Pittsburgh, PA, USA). Approximately 50 mg of *B. atrox* venom were dissolved in 200 µL of elution buffer (20 mM Tris-HCl + 150 mM NaCl + 1 mM CaCl_2_, pH 7.8) and centrifuged at 14,000 *g* for 10 min. After centrifugation, the pellet was discarded and the supernatant was injected into the column HiPrep 16/60 Sephacryl S-200 HR (120 mL 16 × 600 mm, GE Healthcare, Pittsburgh, PA, USA). The parameters for elution were flow rate at 0.5 mL/min, detection system UV_280nm_ and isocratic elution for 260 min. The fractions possessing hemorrhagic and dermonecrotic activities were dialyzed against buffer A (20 mM Tris-HCl + 1 mM CaCl_2_, pH 8.5). After dialysis, samples were manually injected (loop of 2 mL) into an anion exchange column (Mono Q 5/50 GL, 1 mL 5 × 50 mm; GE Healthcare, Pittsburgh, PA, USA) and the elution was performed with buffer A at a flow rate of 1 mL/min, detection system UV_280nm_, with a gradient of 0 to 1 M NaCl, as follows: 0 M NaCl for 6 min, followed by 0 to 0.2 M NaCl over 20 min, 0.2 to 1 M NaCl over 7 min and 1 M NaCl for 7 min. Again the fractions were evaluated for their ability to cause hemorrhage and dermonecrosis and the selected fraction was stored at −80 °C until use.

### 4.4. Protein Identification

The selected protein fraction was analyzed previously SDS-PAGE method as described by [50] using a 12.5% gel, under native or reducing condition by treatment with 2-Mercaptoethanol (58.3 mM). Gels were stained with 0.1% Coomassie Brilliant Blue R-250 and after destaining the gel, the protein band was excised and *in gel* digested with trypsin [51]. For desalinization the sample was loaded in Sep-pak C18 cartridges (Waters, Milford, USA) previously conditioned with 0.1% TFA and eluted with 0.1% TFA in H_2_O/acetonitrile (ACN) (50:50). The resulting peptide eluate was dried using a speedvac concentrator and dissolved in 20 μL of 0.1% formic acid (solution A). Each peptide mixture (5 μL) was injected into a 4 cm C-18 trap column 10 μm Aqua C-18 beads (Phenomenex, Torrance, CA, USA) (100 μm I.D.) coupled to an LTQ-Orbitrap Velos mass spectrometer (Thermo Fisher Scientific, Waltham, MA ,USA). Chromatographic separation of tryptic peptides was performed on 10 cm long column (75 μm I.D.) packed in-house with 5 μm Aqua C-18 beads (Phenomenex, Torrance, CA, USA). Peptides were eluted with a linear gradient of 5–40% acetonitrile in 0.1% formic acid (solution B) at 300 nL/min in 45 min. Spray voltage was set at 2.0 kV and the mass spectrometer was operated in data dependent mode, in which one full MS scan was acquired in the *m*/*z* range of 200–2000 followed by MS/MS acquisition using collision-induced dissociation of the ten most intense ions from the MS scan. MS spectra were acquired in the Orbitrap analyzer at 60,000 resolution (at 400 *m*/*z*) whereas the MS/MS scans were acquired in the linear ion trap. Isolation window, activation time and normalized collision energy were set to, respectively, 2 *m*/*z*, 10 ms and 35%.The search in the database and identification of peptides/proteins was performed using the Mascot program (version 2.4.1; Matrix Science, London, UK) using as database composed of 152 transcript sequences derived from the analysis of venom glands from five adult specimens of *B. atrox* captured at Tapajós National Forest (*n* = 3) and at pasture area in the municipality of Oriximiná (*n* = 2), in Pará State, Brazil (Gene Bank SRA SRP056745); sequences available under accession numbers JAV01810.1 to JAV01961.1. Search parameters were: parent tolerance of 10 ppm and fragment tolerance of 0.5 Da; iodoacetamide derivatives of cysteine and oxidation of methionine were specified in Mascot, respectively, as fixed and variable modifications.

### 4.5. Proteolytic Activity Assays In Vitro

#### 4.5.1. Fluorimetric Assays

The fluorimetric assays were performed using FRET (Fluorescence Resonance Energy Transfer) peptides as described [52] and modified [53]. The FRET peptide used was Abz-AGLA-EDDnp (MM 657.65 Da) from GenOne Biotechnologies (Rio de Janeiro, RJ, Brazil). For assays the concentration of FRET peptide was 200 µM in the reaction buffer (50 mM Tris-HCl, 10 mM CaCl_2_, 150 mM NaCl, 0.05% Brij 35™, pH 7.5). Toxins were diluted with the same buffer in equimolar concentrations. The reactions was carried out at 37 °C and were monitored by measuring fluorescence at λ_EM_ 420 nm and λ_EX_ 320 nm using a spectrofluorimeter (SpectraMax M2, Molecular Devices, Sunnyvale, CA, USA) in kinetics mode. The activity was expressed as mean and standard error (SEM) of relative fluorescence units of three independent experiments. 

#### 4.5.2. Hydrolysis of Fibrin

The hydrolysis of fibrin was accessed according to the method described by [54] and modified by [55]. Samples (50 µL) of isolated SVMPs at different concentrations were applied to small holes made in plates containing a layer of solidified fibrin-agarose gel (2% low melting agarose; Amresco^®^, Solon, OH, USA), 3 mg/mL of bovine fibrinogen (Sigma-Aldrich, St. Louis, MO, USA) and 2 U/mL of thrombin (Sigma-Aldrich, St. Louis, MO, USA) in 50 mM Tris–HCl containing 200 mM NaCl, 50 mM CaCl_2_, pH 7.3. Plates were incubated at 37 °C for 18 h and the hydrolysis halo was obtained by subtracting the area of lysis from the area of the sample well. Results are expressed as mean and standard error (SEM) of the area of lysis (cm^2^) from three independent experiments.

#### 4.5.3. Hydrolysis of Proteins of the Basement Membrane

Proteolysis of the BM components was evaluated as described [12], with some modifications. The SVMPs were incubated with Matrigel (Sigma-Aldrich, St. Louis, MO, USA), a reconstituted BM extracted from the Engelbreth-Holm-Swarm mouse sarcoma, at an enzyme/substrate ratio of 0.5:10 and 1:10 (*w*:*w*); incubations were maintained for 30 min, 1 h and 3 h, at 37 °C. Matrigel incubated without SVMPs was used as control. Reactions were stopped by addition of SDS-PAGE sample buffer, with reducing agent (58.3 mM of 2-Mercaptoethanol), boiled for 5 min and analyzed by SDS-PAGE and Western Blotting. 

For SDS-PAGE, contents of the reaction mixture were separated using a 5 to 15% gradient gel under reducing conditions. Gels were stained with 0.1% Coomassie Brilliant Blue R-250 and the molecular masses calculated by comparison with the rainbow molecular mass standards (GE Healthcare, Pittsburgh, PA, USA). For Western Blotting, after SDS-PAGE the proteins were electro-transferred to nitrocellulose membranes for 1 h (30 V and 60 mA). Membranes were blocked for 2 h with 5% low-fat milk in TBS (Tris-buffered saline) and incubated overnight at 4 °C with 0.35 µg/mL rabbit polyclonal anti-laminin (Novus Biologicals, Littleton, CO, USA), 10 µg/mL rabbit polyclonal anti-collagen IV (Fitzgerald, Acton, MA , USA), or 0.6 µg/mL goat polyclonal anti-nidogen 1(R&D Systems, Minneapolis, MN, USA). After incubation the membranes were washed with TBS and incubated with peroxidase-conjugated anti-rabbit IgG (1:1000) or anti-goat IgG (1:250) (Sigma-Aldrich, St. Louis, MO, USA) at room temperature for 1 h and the reactive bands were detected by incubation with 4-chloro-a-naphthol (Sigma-Aldrich, St. Louis, MO, USA) and H_2_O_2_. 

### 4.6. Binding of SVMPs to Plasma and Extracellular Matrix Proteins In Vitro

The adhesion of SVMPs with fibrinogen, fibronectin, collagens I and IV, laminin and nidogen was estimated according to the method reported previously [39] with some modifications. Microtiter plates (Costar^®^ High Binding/Corning, Corning, NY, USA) were coated with collagens I and IV, laminin, fibrinogen, fibronectin (10 µg/mL) and nidogen (5 µg/mL) in carbonate-bicarbonate buffer (pH 9.6), and incubated for 18 h at 4 °C. Plates were washed with TBS and incubated for 2 h with 2% low-fat milk in TBS at 37 °C. Different dilutions of toxins were added to the wells and incubated for 1 h at 37 °C, followed by washing and an additional incubation with mouse anti-Batroxrhagin serum at a dilution of 1:100 for 1 h at 37 °C. After washing, plates were incubated with peroxidase-conjugated anti-mouse IgG (Sigma-Aldrich, St. Louis, MO, USA) at a dilution of 1:2000 for 1 h at 37 °C. Reaction were revealed by adding 15 mg/mL of O-Phenylenediamine (Sigma-Aldrich, St. Louis, MO, USA) containing 0.06% of H_2_O_2_ and stopped with 30% H_2_SO_4_. The binding was detected at 492 nm in a plate reader (SpectraMax M2, Molecular Devices, Sunnyvale, CA, USA) and results are expressed as mean and SEM of the reading from three independent experiments. 

### 4.7. Biological Activities In Vivo

#### Hemorrhagic and Dermonecrotic Activities

The activities were determined according on the method previously described [56] with some modifications. Different concentrations of the isolated PI-class SVMP in 50 µL of phosphate-buffered saline (PBS) were injected intradermally into the dorsal skin of mice. The animals were euthanized in a CO_2_ chamber at 20 min, 3, 6 and 24 h after an injection and the dorsal skin was removed, digitized and saved in RGB (Red, Green and Blue) format files for processing in a Matlab script [57] using the procedure described previously [58]. The different components of the image were selected using a threshold device [59] and a morphological gradient processor to obtain the hemorrhagic area. The area was measured using the approach of Dougherty and Lotufo [60]. The minimum hemorrhagic dose (MHD) was defined as the protein dose (µg) that produced hemorrhages with a mean diameter of 10 mm at 3 h after injection. The minimum necrotizing dose (MND) was considered as the least amount of toxin in µg injected intradermally that resulted in a necrotic lesion of 5 mm diameter 24 h after injection. Groups of 3 animals were tested in three independent experiments for the determination of MHD and groups of 4 animals were tested for the determination of MND. The hemorrhage and dermonecrosis were also investigated with SVMPs pre-incubated for 30 min at 37 °C with PMSF (20 mM of Phenylmethylsulfonyl fluoride diluted in ethanol) or O-Phe (20 mM of 1,10-Phenanthroline diluted in dimethyl sulfoxide). Inhibition of activities was analyzed at 3 h after the injection and groups of 4 animals were used.

### 4.8. Histological Analysis and Detection of ECM Components

Histological analysis of the lesions induced by toxins was carried out after injection of 10 µg of pure toxins or conjugated with Alexa Fluor 488 for immunodetection of toxins in the tissue. PBS or Alexa Fluor 488 conjugated BSA (bovine serum albumin) were used as controls. The animals were euthanized at 20 min or 24 h after the injection and the dorsal skin corresponding to the site of the injection was carefully dissected out and subjected to paraffinization or frozen in OCT (Optimal cutting temperature). After paraffinization sections of 5 mm were adhered to glass slides using 0.1% poly-l-Lysine (Sigma-Aldrich, St. Louis, MO, USA) and dried at room temperature. The histological sections were dewaxed in xylol, hydrated in distilled water and staining the sections using hematoxylin and eosin staining for general tissue inspection. After this process, the slides were subjected to dehydration, diaphanization and assembly. 

The distribution of toxins and distribution of basement membrane components was analysed by immunofluorescence. Sections (5 mm) from frozen tissues were adhered to glass slides using 0.1% poly-l-Lysine and fixed in 3.7% formaldehyde for 15 min at room temperature. The sections were blocked by incubating for 2 h at room temperature with PBS containing 1% triton X-100, 5% normal goat serum, 1% BSA, 0.5% glycine and 0.5% fish skin gelatin. After this, sections were incubated with rabbit polyclonal anti-laminin (Novus Biologicals, Littleton CO, USA), 10 µg/mL rabbit polyclonal anti-collagen IV (Fitzgerald, Acton, MA, USA) for 18 h at 4 °C. The sections were washed with PBS and incubated with TRITC-labeled (Tetramethyl Rhodamine Isothiocyanate) goat anti-rabbit IgG (Jackson ImmunoResearch, West Grove, PA, USA) and 4′,6-diamidino-2-phenylindole (DAPI) (Molecular Probes, Thermo Fisher Scientific, Waltham, MA, USA), at a 1:100 and 1:5000 dilution respectively, for 90 min at room temperature. The sections were examined at least 10 different fields with a Confocal Microscope Zeiss LSM 780-NLO (Zeiss, Oberkochen, Germany). 

### 4.9. Statistical Analysis

The results are expressed as the mean and standard error (SEM) of the mean. Analysis of Variance (ANOVA) One-Way followed by Tukey post-test (for multiple comparisons) were used for comparison of means. The level of significance was set at *p* ≤ 0.05.

## Figures and Tables

**Table 1:** Identification of the peptides generated by *in gel* trypsin digestion of protein band shown in Figure 1B by *.

Identified Protein #	Mascot Protein Score	Expect	Identified Peptides
BATXSVMPI5	4966	1.4 ×10^−3^	TDLLNR
		9.8 × 10^−4^	KTDLLNR
		1.3 × 10^−1^	TDLLNRK
		3.5 × 10^−2^	KTDLLNRK
		1.5 × 10^−4^	IHQMVNIMK
		1.4 × 10^−4^	ENPQCILNK
		2.0 × 10^−4^	RIHQMVNIMK
		1.7 × 10^−5^	ENPQCILNKR
		5 × 10^−8^	STGVIQDHSEQDLK
		9.2 × 10^−5^	RSTGVIQDHSEQDLK
		8.3 × 10^−5^	YVELLIVVDHGMFMK
		5.9 × 10^−6^	YFSDCSYIQCWDFIMK
		4.9 × 10^−13^	DLINVQPAAPQTLDSFGEWR
		9.6 × 10^−7^	DLINVQPAAPQTLDSFGEWRK
		9.3 × 10^−6^	EAYSTMYIDILLTGVEIWSNK
		1.3 × 10^−6^	SHDNAQLLTSTDFNGPTIGLAYVGSMCDPK
		9.2 × 10^−6^	KSHDNAQLLTSTDFNGPTIGLAYVGSMCDPK
		2.8 × 10^−11^	SHDNAQLLTSTDFNGPTIGLAYVGSMCDPKR
		3.3 × 10^−6^	KSHDNAQLLTSTDFNGPTIGLAYVGSMCDPKR
		1.0 × 10^−3^	VAITMAHELGHNLGISHDTGSCSCGGYSCIMSPVLSHEPSK
		4.0 × 10^−2^	EAYSTMYIDILLTGVEIWSNKDLINVQPAAPQTLDSFGEWR

# Amino acid sequences were identified using a *B. atrox* venom gland transcriptome database, described in Methods section.
